# Comprehensive Safety and Efficacy Evaluation of Immunotherapy Combination Approaches Versus Tyrosine Kinase Inhibitor Monotherapy as First-Line Treatment of Hepatocellular Carcinoma: A Network and Individual Patient Data (IPD) Meta-Analysis

**DOI:** 10.3390/cancers18132118

**Published:** 2026-06-30

**Authors:** Abdullah Esmail, Yazan Hamdaneh, Nour Mustafa, Ebtesam Al-Najjar, Zaid Alabed, Hikmat Abdel-Razeq, Asem Mansour, Maen Abdelrahim

**Affiliations:** 1 Section of GI Oncology, Department of Medicine, Houston Methodist Cancer Center, Houston, TX 77030, USA; aesmail@houstonmethodist.org; 2 Department of Medicine, Weill Cornell Medical College, New York, NY 10065, USA; 3 Faculty of Medicine, The University of Jordan, Amman 11942, Jordan; 4 Department of Internal Medicine, King Hussein Cancer Center, Amman 11941, Jordan; 5 Department of Radiology, King Hussein Cancer Center, Amman 11941, Jordan

**Keywords:** immunotherapy, tyrosine kinase inhibitor, hepatocellular carcinoma, individual patient data (IPD) meta-analysis

## Abstract

**Background**: Hepatocellular carcinoma (HCC) represents the most common primary liver cancer and a significant cause of global cancer-related mortality, with the majority of patients diagnosed at advanced or unresectable stages. Historically, tyrosine kinase inhibitor (TKI) monotherapies such as sorafenib and lenvatinib served as the primary systemic standard of care. However, the emergence of immune checkpoint inhibitor (ICPI)-based combinations has significantly transformed the treatment landscape. We aim to perform a comparative analysis of ICPI-based combination treatment versus TKI monotherapy among advanced HCC patients. **Methods**: This study utilized a reconstructed individual patient data (IPD) pooled analysis derived from nine phase 3 randomized clinical trials, adhering to PRISMA-IPD reporting guidelines. A total of 6161 patients were included in the analysis, which categorized treatment into five primary strategies: ICPI monotherapy, ICPI plus bevacizumab, dual ICPI therapy (duplet), ICPI plus TKI, and TKI monotherapy as the control group. The primary endpoint was overall survival (OS), with secondary endpoints including progression-free survival (PFS) and the incidence of grade 3 or higher adverse events (AEs). **Results**: The analysis demonstrated that ICPI-based combinations provided a significant survival advantage over TKI monotherapy. Key hazard ratios (HRs) for OS compared to TKIs were 0.76 (95% CI: 0.67–0.85, *p* < 0.001) for dual ICPI, 0.76 (95% CI: 0.68–0.85, *p* < 0.001) for ICPI plus TKI, and 0.70 (95% CI: 0.61–0.81, *p* < 0.001) for ICPI plus bevacizumab. Median OS was numerically highest for ICPI plus TKI at 19.68 months and dual ICPI at 19.58 months compared to 14.84 months for the TKI group. Among FDA-approved regimens, nivolumab plus ipilimumab (NivoIpi) achieved the longest median OS of 24.08 months. From a safety standpoint, the ICPI plus TKI group had the highest incidence of grade 3/4 AEs at 69.1%. Conversely, TKI monotherapy showed a 50.1% incidence, while dual ICPI therapy exhibited the most favorable safety profile at 32.7%. **Conclusions**: ICPI-containing combination therapies are superior to TKI monotherapy for the first-line treatment of advanced HCC, providing marked improvements in survival outcomes. Dual ICPI therapy represents the most balanced approach between efficacy and safety, achieving high survival with the lowest rates of severe toxicity. Among approved options, NivoIpi exhibited a numerically favorable survival signal, while DurvaTreme offered the highest tolerability, supporting a personalized treatment approach based on individual patient risk factors and hepatic reserve.

## Simple Summary

Advanced hepatocellular carcinoma is a premier contributor to global oncology mortality, with therapeutic standards historically restricted to tyrosine kinase inhibitor monotherapy. Although the introduction of diverse immune checkpoint inhibitor combinations has shifted the frontline treatment paradigm, a clinical knowledge gap remains regarding the comparative efficacy and safety across these distinct programmatic strategies due to a lack of head-to-head clinical trials. This study aims to evaluate these frontline regimens by reconstructing individual patient data from nine phase 3 randomized controlled trials. The findings demonstrate that immunotherapy-based combinations significantly prolong survival compared to tyrosine kinase inhibitors, with distinct regimens offering varying balances of therapeutic efficacy and severe toxicity. This study impacts the GI oncology community by providing a robust framework to guide personalized clinical decision making, allowing clinicians to tailor systemic therapy selection to an individual patient’s baseline hepatic reserve and programmatic risk profile.

## 1. Introduction

Hepatocellular carcinoma (HCC) is the most common primary liver cancer and one of the leading causes of cancer-related mortality worldwide [1]. According to the Global Cancer Statistics 2020 report, liver cancer is the sixth most commonly diagnosed cancer and the third leading cause of cancer-related death worldwide, accounting for approximately 906,000 new cases and 830,000 deaths annually [2,3,4]. In the United States (US), liver cancer ranks as the fifth leading cause of cancer-related mortality, with an estimated 42,340 new cases and 30,980 deaths annually [5].

It is strongly associated with liver cirrhosis, hepatitis B/C infections, and fatty liver diseases [6]. In addition, metabolic dysfunction-associated steatotic liver disease (MASLD) has emerged as an increasingly important contributor to the global burden of HCC, with a growing number of cases occurring in patients with metabolic risk factors such as obesity, diabetes, and metabolic syndrome [7].

HCC has a favorable prognosis when it is discovered and treated in its early stages [4]. However, most patients are diagnosed with advanced disease, making surgical resection an unfeasible choice. Unresectable hepatocellular carcinoma (uHCC) is characterized by multiple tumors, large masses (more than 5 cm), macrovascular invasion, liver dysfunction, or metastasis [8]. These patients often exhibit macrovascular invasion or extrahepatic metastasis, highlighting the urgent need to identify effective and tolerable therapies with high prognostic value.

The Barcelona Clinic Liver Cancer (BCLC) staging system is the most widely adopted framework for HCC management and provides treatment recommendations based on tumor stage, liver function, and performance status, ranging from curative therapies for early-stage disease to systemic treatment for advanced disease [9].

Management of HCC should ideally be performed within a multidisciplinary framework involving hepatologists, oncologists, surgeons, interventional radiologists, and transplant specialists to optimize treatment selection and outcomes.

The treatment of HCC is particularly complex because clinical outcomes are influenced not only by tumor characteristics but also by the degree of underlying liver dysfunction. Consequently, therapeutic decision making requires careful consideration of disease stage, hepatic reserve, and patient performance status to achieve an optimal balance between efficacy and safety [9].

Historically, the treatment options for advanced HCC were limited, with locoregional therapy such as transcatheter arterial chemoembolization (TACE) and targeted therapy including sorafenib, a multikinase inhibitor targeting vascular endothelial growth factor receptors (VEGFR) and RAF pathways, and lenvatinib, a multikinase inhibitor targeting VEGFR and fibroblast growth factor receptors (FGFR), providing modest survival benefits compared with previous chemotherapy [10,11,12,13,14,15]. Following the immunotherapy revolution, various treatment combinations were used as first-line therapy for uHCC. The Imbrave150 trial established atezolizumab, a programmed death-ligand 1 (PD-L1) inhibitor, combined with bevacizumab, a vascular endothelial growth factor (VEGF) inhibitor, as a first-line standard of care, demonstrating significant improvements in overall survival (OS) and progression-free survival (PFS) compared with sorafenib [16].

As cancer management continues to evolve, there is increasing interest in comprehensive treatment approaches that integrate advances in systemic therapy, supportive care, nutritional interventions, and other evidence-based strategies to optimize patient outcomes and quality of life. Recent reviews have highlighted the importance of continuously reassessing emerging therapeutic approaches as new scientific and clinical evidence becomes available [17,18].

More recently, multiple immunotherapy-based combinations have shown promising results [12,19]. Dual immune checkpoint inhibitors (ICPIs), such as durvalumab, a PD-L1 inhibitor, combined with tremelimumab, a cytotoxic T-lymphocyte-associated antigen 4 (CTLA-4) inhibitor (HIMALAYA trial), and nivolumab plus ipilimumab (CheckMate-9DW trial), have demonstrated improved OS compared with standard therapies [20,21,22].

In addition, combinations of ICPIs with antiangiogenic agents or tyrosine kinase inhibitors (TKIs), including camrelizumab (PD-1 inhibitor) plus rivoceranib (VEGFR-2 inhibitor) in the CARES-310 trial, anlotinib (multikinase inhibitor) plus penpulimab (PD-1 inhibitor) in the APOLLO trial, toripalimab (PD-1 inhibitor) plus bevacizumab (VEGF inhibitor) in the HEPATORCH trial, and sintilimab (PD-1 inhibitor) plus bevacizumab biosimilar in the ORIENT-32 trial, have consistently demonstrated significant improvements in survival outcomes compared with sorafenib [23,24,25,26].

Despite these rapid advances and the evolving first-line standards in clinical guidelines like the NCCN, no head-to-head comparisons currently exist among these diverse regimens. This creates significant ambiguity in selecting the optimal strategy for individual patients. Notably, this study is among the first network meta-analyses to utilize reconstructed individual patient data (IPD) to integrate recent landmark trials, including CheckMate-9DW and HIMALAYA. Network meta-analysis using reconstructed IPD from Kaplan–Meier survival curves provides a robust method to indirectly compare multiple treatments and evaluate their relative efficacy and safety.

Therefore, in this systematic review and network meta-analysis, we aim to compare the efficacy and safety of the current first-line treatment options in patients with uHCC using reconstructed IPD from Kaplan–Meier survival curves.

## 2. Methodology

### 2.1. Study Design and Search Strategy

A reconstructed IPD pooled analysis of phase 3 randomized clinical trials was performed on selected studies following the Preferred Reporting Items for Systematic Reviews and Meta-analyses (PRISMA) and reporting guidelines for IPD (PRISMA-IPD).

A systematic review was conducted on PubMed, Scopus, and Embase using the following search protocol: “((Hepatocellular carcinoma or HCC) and (First-line treatment) AND ((atezolizumab OR bevacizumab OR nivolumab OR ipilimumab OR durvalumab OR tremelimumab OR lenvatinib OR sorafenib)))”. Studies up until July 2025 were included in our meta-analysis. Studies that meet the following criteria were selected: (1) phase 3 randomized clinical trials; (2) patients with metastatic or non-resectable HCC; (3) adult patients who are older than 18 years; (4) any trial group that includes novel first-line treatment or currently approved first-line treatment; and (5) available Kaplan–Meier plots with number at risk tables for OS and PFS.

### 2.2. Data Extraction and Risk of Bias

Two investigators (N.M. and E.A.) independently reviewed the titles and abstracts of all identified studies. Eligible studies underwent a two-stage screening process performed independently by A.E. and Z.A., consisting of title and abstract screening followed by full-text review. Disagreements regarding study inclusion were resolved by a third independent reviewer (A.E.). The reviewers N.M. and E.A. independently extracted the following data from the included studies: study identification (journal name, publication date, countries, and ethics approval status), patient characteristics (sample size, age, and gender), interventions (atezolizumab plus bevacizumab, nivolumab plus ipilimumab, durvalumab plus tremelimumab, durvalumab, camrelizumab plus rivoceranib, anlotinib plus penpulimab, toripalimab plus bevacizumab, sintilimab plus bevacizumab, lenvatinib plus pembrolizumab, and lenvatinib monotherapy), comparator (sorafenib or lenvatinib), and outcomes (overall survival, progression-free survival, and safety). Only the most updated and complete publication was used as a trial source for studies with multiple publications. The trials were assessed for risk of bias using the Cochrane Risk of Bias (version 2) tool for randomized clinical trials (RCTs) [27].

### 2.3. Time to Event Outcomes Reconstruction

Time to event outcomes (OS and PFS) were estimated using a web tool for graphical reconstruction from reported Kaplan–Meier plots for each group of each study using a validated web-based digitization tool. For each curve, coordinates were sampled at multiple time points, incorporating information on the numbers at risk and censoring where available, this approach was guided by previous published studies by Zhao JJ et al., Nichetti F et al., Raimondi et al., and Pietrantonio et al., based on this method developed by Guyot et al. and implemented by Liu et al. The data reconstruction was performed independently by two investigators (E.A and N.M), and the best reconstruction was selected after the data was cross checked against the published hazard ratio. The potential sources of errors with this approach include limited image resolution and manual digitization variability, but these were minimized by independently duplicate the extraction and systematic comparison. IPD of the same treatment groups from different trials were pooled [28,29,30,31,32,33].

### 2.4. Data Analysis

The primary endpoint for the analysis was OS, defined as the duration from the treatment initiation to the last follow-up or death within the range of the observation period in the clinical trials for each of the treatment groups. Secondary end points were PFS, defined as time between treatment initiation and progression, death, or last follow-up within the range of observation periods in the clinical trials for each of the treatment groups, and the rate of grade 3 or higher treatment-related adverse events (AEs) for each group. Studies not reporting a specific adverse event (AE) were excluded from their respective analysis.

Pooled PFS and OS curves were estimated with the Kaplan–Meier method, while the reverse Kaplan–Meier estimator was used to quantify the median follow-up. All were compared using global log-rank tests. The outcome for each arm was studied using Cox proportional hazards regression models, with individual patient’s trial data included as a random variable to account for interstudy differences as shown by previous works Nichetti et al., Pietrantonio et al., and Raimondi et al. Tukey’s test for pairwise test was used to conduct pairwise log-rank tests for survival and between different experimental arms [29,30,31].

Treatment arms were categorized and pooled into five groups. TKIs, which consists of sorafenib and lenvatinib arms, were chosen as the control arm. ICPI dual therapy, including nivolumab plus ipilimumab (NivoIpi) and durvalumab plus tremelimumab (DurvaTreme); ICPI monotherapy, including a durvalumab arm; ICPI plus bevacizumab, including atezolizumab plus bevacizumab (AtezoBev), toripalimab plus bevacizumab (ToriBev), and sintilimab plus bevacizumab (SintiBev); and ICPI plus TKIs, including pembrolizumab plus lenvatinib (LenvaPembro), camrelizumab plus rivoceranib (CamreRivo) and anlotinib plus penpulimab (AnloPenp) were chosen as experimental groups. These categories were chosen to reflect the regimens which commonly use in clinical practice to ensure adequate event numbers for stable estimations. Additionally, further analysis was done on these Food and Drug Administration (FDA) approved first-line combinations (DurvaTreme, NivoIpi, and AtezoBev).

To validate the results of the pooled analysis, a frequentist method-based network meta-analysis was conducted using the hazard ratios extracted from the original trials.

Moreover, to show that our OS and PFS findings are significant, power and potential sample size analyses were conducted using the estimated treatment effect size from the Cox proportional hazards models of the derived subgroups. Specific drug experimental subgroups and class experimental subgroups were compared to a pooled control (TKI) subgroup, and the study power was evaluated using the hazard ratios (HRs) of the comparison group with alpha = 0.05. Sensitivity analyses were also performed including restriction to FDA-approved first line combinations and their TKI control and exploration of alternative treatment class groupings. In case of similar outcomes between two regimens (e.g., HRs between 0.90 and 1.10), a non-inferiority design was adopted.

Grade 3 or higher treatment-related AEs were pooled using single-proportion meta-analysis and compared with χ^2^ tests. Statistical heterogeneity across studies was assessed using the I^2^ statistic and Cochran’s Q test.

Here, *p* = 0.05 was set as the statistical significance threshold, and all statistical tests were two sided. The analysis was conducted using R software version 4.4.2 (R Foundation for Statistical Computing). A list of R packages used for the analysis is provided in Supplemental S1.

## 3. Results

### 3.1. Study Selection and Characteristics

**Figure 1:**
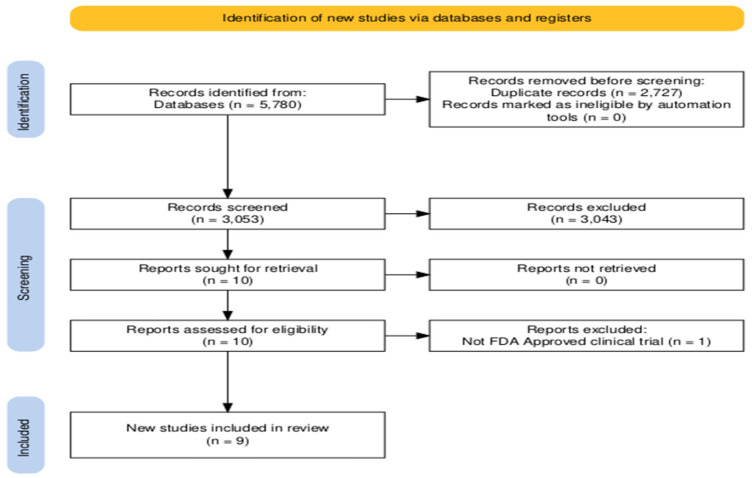
PRISMA flowchart of included studies.

A total of 5780 studies went through title/abstract screening, and nine studies were included in the analysis as seen in Figure 1. The main analysis included 6161 patients. In total, 3066 (50%) were in the control arm, while 3095 (50%) patients were included in the experimental arm. Patient numbers across different treatments, classes, and trials are shown in Table S1 of Supplemental S1.

All trials had either sorafenib or lenvatinib as a control arm except CheckMate 9DW, which had a control arm consisting of a combination of patients treated with sorafenib or lenvatinib. However, supplementary OS Kaplan–Meier curves separated the patients, and those curves were used to reconstruct patient data for their respective treatments. The merged arm was used to extract PFS data from the trial. PFS data were not used in the specific treatment analysis, only in the classes analysis. The characteristics of the trials are shown in Table 1. Risk of bias analysis resulted in low bias across all included studies as shown in Figure S1 of Supplemental S1 and Supplemental S2.

### 3.2. Survival Outcomes

**Figure 2:**
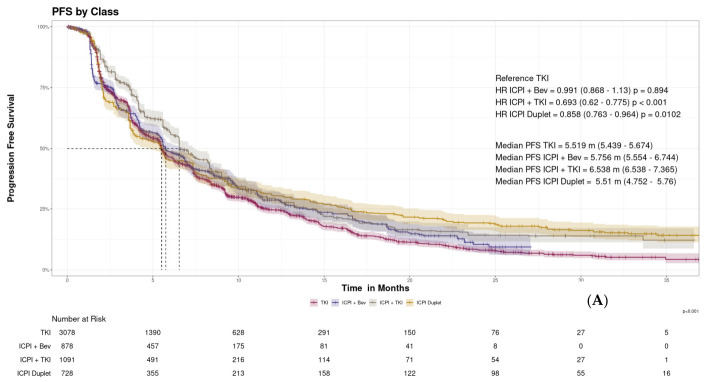

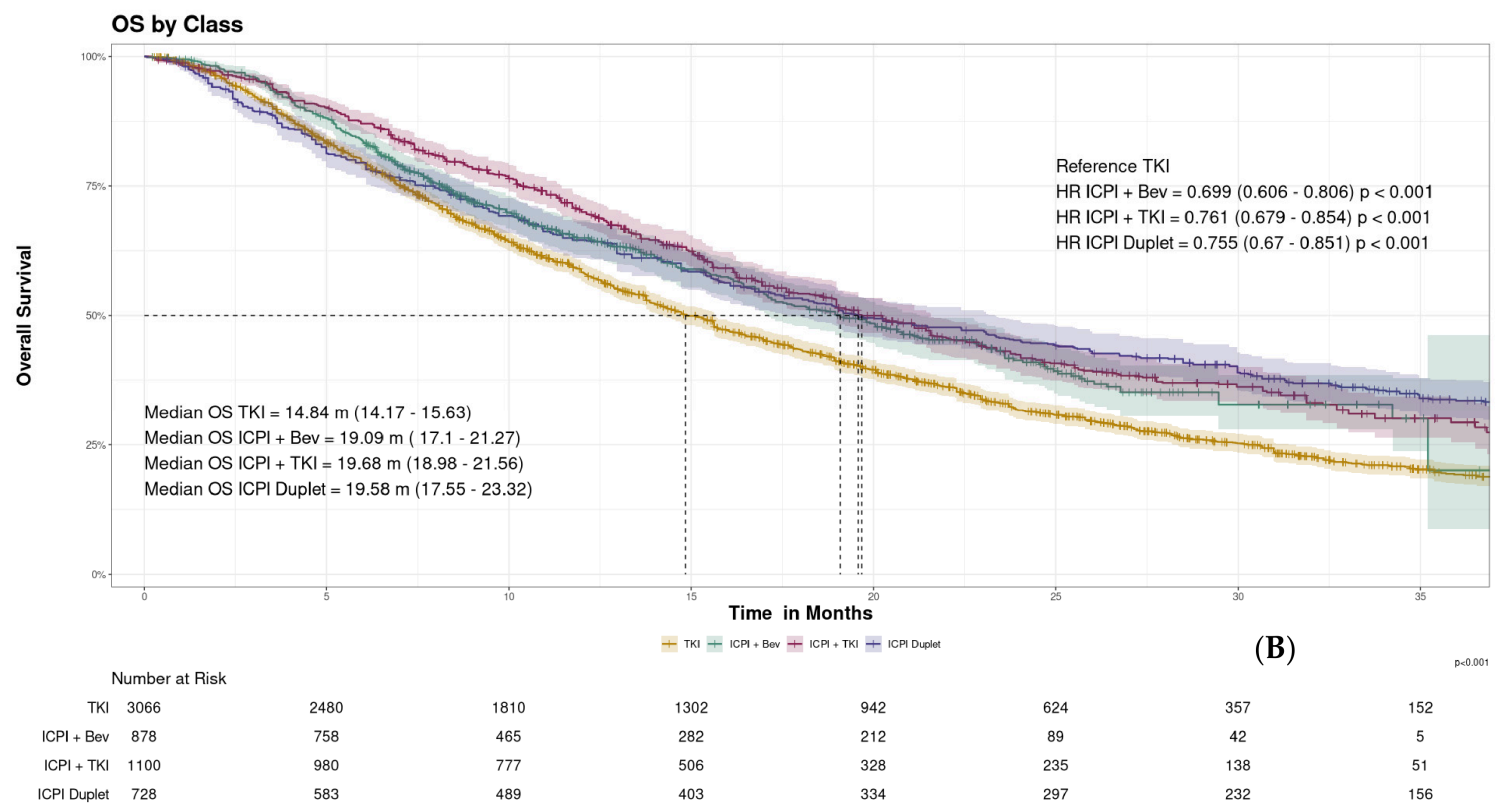
Kaplan–Meier curves comparing (**A**) PFS of patients who received different classes of drugs compared to TKIs and (**B**) OS of patients who received different classes of drugs compared to TKIs.

Overall median progression-free survival (mPFS) was 5.59 months (95% CI: 5.52–5.72). ICPI plus TKI achieved the highest mPFS at 6.54 months (95% CI: 6.36–7.37), followed by ICPI plus bevacizumab at 5.76 months (95% CI 5.55–6.74), dual ICPI at 5.51 months (95% CI: 4.75–5.76), TKI monotherapy at 5.52 months (95% CI: 4.77–5.52), and ICPI monotherapy at 3.73 months (95% CI: 3.35–3.89) (Figure 2A), with a global *p*-value <0.001.

Median overall survival (mOS) was 16.95 months (95% CI 16.24–17.61). Dual ICPI therapy and ICPI plus TKI achieved the numerically highest mOS at 19.58 months (95% CI: 17.56–23.32) and 19.68 months (95% CI 18.99–21.56), respectively, and ICPI plus bevacizumab at 19.09 months (95% CI: 17.10–21.27). ICPI monotherapy yielded a mOS of 16.84 months, while TKI monotherapy demonstrated the lowest OS at 14.84 months (95% CI: 13.17–15.63) (Figure 2B), with a global *p*-value <0.001.

As shown in Table 2, ICPI + TKI and ICPI duplet showed better PFS and OS outcomes compared to control, while ICPI + Bev showed only significantly better OS compared to TKI control. There were no significant differences in OS and PFS between ICPI monotherapy and control.

Pairwise analysis showed no significant difference in OS between ICPI + Bev, ICPI + TKI, ICPI duplet and ICPI monotherapy arms. However, ICPI + Bev and ICPI monotherapy had worse PFS compared to ICPI + TKI (HR = 1.43, 95% CI 1.12–1.81, *p* < 0.001) and (HR = 1.39, 95% CI 1.08–1.78, *p* = 0.003). There was no significant difference in PFS between ICPI + TKI and ICPI duplet (HR = 0.81, 95% CI 0.64–1.01, *p* = 0.61).

### 3.3. Safety Analysis

From a safety standpoint, the overall incidence of grade III/IV AEs was highest in the ICPI plus TKI group (69.1%), followed by TKI monotherapy (50.1%) and ICPI + Bev (38.6%). Notably, ICPI + TKI and TKI monotherapy had the highest rates of hypertension, increased bilirubin levels, and diarrhea. In contrast, anemia was more common in the ICPI + Bev group. The prevalence of the most commonly reported adverse events are summarized in Table 3 and Table S2 in Supplementary S1.

### 3.4. Head-to-Head Comparison of FDA First Line Combinations

Of the 6161 patients, 1064 (17.3%) patients received FDA-approved first-line combinations. In total, 393 patients received DurvaTreme, 336 received AtezoBev, and 335 received NivoIpi. In total, 1146 patients who received lenvatinib were included in the analysis as controls.

As shown in Table 4 and Table 5, NivoIpi had the numerically longest mOS and mPFS, and it showed superior outcomes compared to lenvatinib (HR OS 0.70 95% CI 0.60–0.82, *p* < 0.001 and HR PFS 0.73 95% CI 0.63–0.85, *p* < 0.001). However, there was no significant difference between the OS of AtezoBev and DurvaTreme compared to control.

Grade 3/4 adverse events were highest with lenvatinib (66.3%) and lowest with DurvaTreme (25.5%) compared with AtezoBev (42.6%) and NivoIpi (40.9%) (*p* < 0.001). Most notably, lenvatinib had the highest rates of grade 3/4 fatigue (2.6%), hand-foot skin reaction (3.1%), decreased appetite (3.9%), and hypertension (20.3%). Moreover, the most common adverse event for AtezoBev was hypertension (12%). Details about the prevalence of different grade 3/4 adverse events is shown in Table S3 of Supplemental S1.

## 4. Discussion

HCC remains among the most lethal malignancies worldwide [2]. The majority of patients are diagnosed at an advanced, unresectable stage, limiting potentially curative treatment options. For over a decade, sorafenib and lenvatinib, both of which are TKIs, served as the standard of care for first-line systemic therapy, offering modest median OS of approximately 10–14 months [4,6]. The emergence of ICPI-based combination strategies has started to largely transform the treatment landscape, introducing regimens that significantly outperform TKI monotherapy in randomized clinical trials. This study provides a comprehensive and quantitative synthesis of IPD reconstructed from published Kaplan–Meier curves across phase 3 randomized controlled trials, comparing five first-line treatment strategies, including ICPI monotherapy, ICPI plus bevacizumab, ICPI duplet, ICPI plus TKI, and TKI monotherapy, across 6161 patients. Our analysis encompasses multiple subgroup comparisons, each offering distinct and clinically meaningful insights.

### 4.1. Overall Comparative Efficacy Across All Treatment Strategies

The overarching analysis across all five treatment arms incorporating 6161 patients demonstrated statistically significant differences in both OS and PFS (*p* < 0.001). Dual ICPI therapy was associated with numerically higher median OS at 19.58 months (95% CI: 17.56–23.32), followed closely by ICPI plus TKI at 19.68 months (95% CI: 18.99–21.56) and ICPI plus bevacizumab at 19.09 months (95% CI: 17.10–21.27). ICPI monotherapy yielded a median OS of 16.84 months, while TKI monotherapy was associated with the lowest OS at 14.84 months (95% CI: 13.96–16.27). A broadly consistent pattern was observed for PFS, with ICPI plus TKI achieving a higher median PFS at 6.54 months, followed by ICPI plus bevacizumab (5.75 months), dual ICPI (5.51 months), TKI monotherapy (5.52 months), and ICPI monotherapy (3.73 months).

These findings confirm that any ICPI-containing regimen confers a survival advantage over TKI monotherapy alone, consistent with data from individual landmark trials such as IMbrave150, HIMALAYA, CheckMate 9DW, and CARES-310 [16,20,21,23]. The survival advantage conferred by ICPI-containing combinations over TKI monotherapy supports the broad adoption of immunotherapy as the backbone of first-line therapy in eligible patients with advanced HCC.

To be more specific, these results align in part with the IMbrave150 trial that demonstrated that AtezoBev reduced the risk of death by 42% compared to sorafenib (HR 0.58) [16]. The inclusion of real-world data and updated trial analyses further validates these findings in broader patient populations.

From a safety standpoint, the overall incidence of grade III/IV AEs was highest in the ICPI plus TKI group (69.1%), followed by TKI monotherapy (50.1%) and ICPI plus bevacizumab (38.65%). Notably, hypertension (23% versus ~8%), diarrhea (2.84%), and platelet abnormalities (7.8%) were significantly more prevalent in the ICPI plus TKI arm (*p* < 0.001 for platelet abnormalities), while anemia was more frequent with ICPI plus bevacizumab (5.6%, *p* = 0.0275). These findings underscore the variable tolerability profiles of different combination strategies and highlight the importance of individualized patient selection.

### 4.2. Dual ICPI Versus ICPI Plus TKI and TKI Monotherapy

A focused analysis comparing dual ICPI, ICPI plus TKI, and TKI monotherapy revealed that dual ICPI was associated with the longest median OS (19.58 months) alongside the most favorable toxicity profile among the three arms. Grade 3/4 AEs were most frequent with ICPI plus TKI (69.1%) compared to TKI monotherapy (50.1%) and dual ICPI (32.7%), with a trend toward significance (*p* = 0.055). The low rate of severe toxicity in the dual ICPI arm, despite strong antitumor activity, is a particularly important finding, suggesting that the immunological synergy between co-inhibitory pathway blockade, such as NivoIpi (PD-1 and CTLA-4 inhibition), can achieve durable tumor control with an acceptable safety profile.

Mechanistically, the dual blockade of the PD-1/PD-L1 and CTLA-4 pathways targets complementary immune regulatory checkpoints, enhancing T-cell priming and effector function while relieving immunosuppression within the tumor microenvironment [36]. In contrast, the addition of TKIs to ICPIs may amplify toxicity through overlapping AE profiles, particularly hepatotoxicity, hypertension, and gastrointestinal toxicity, without proportional increments in survival benefit. Bilirubin elevation was notably higher in the dual ICPI group, which may reflect immune-mediated hepatitis, a known class effect of CTLA-4 inhibitors that requires close monitoring and appropriate immunosuppressive management [37]. Taken together, these data suggest that dual ICPI represents a highly competitive option in terms of the efficacy–safety balance, particularly for patients with preserved hepatic function.

### 4.3. Dual ICPI Versus ICPI Plus Bevacizumab Versus TKI Monotherapy

The three-arm comparison among dual ICPI (*n* = 728), ICPI plus bevacizumab (*n* = 878), and TKI monotherapy (*n* = 1729) yielded median OS values of 19.58, 19.09, and 15.29 months, respectively (*p* < 0.001), and median PFS values of 5.51, 5.76, and 5.43 months, respectively. While the dual ICPI arm conferred a numerically superior OS, the bevacizumab-containing arm demonstrated marginally higher PFS. This may be attributed to the antiangiogenic mechanism of bevacizumab, which can elicit early tumor stabilization.

Bevacizumab, a monoclonal antibody targeting vascular endothelial growth factor A (VEGF-A), is hypothesized to augment ICPI activity by normalizing the tumor vasculature and decreasing the proliferation and total number of regulatory T-cells (Tregs) via the VEGFR-2 receptor, which effectively reduces the tumor’s immune evasive shield [16]. This immunological rationale can also explain the efficacy of AtezoBev in IMbrave150. Safety analysis in this subgroup revealed a higher incidence of platelet abnormalities with ICPI plus bevacizumab (5.45%, *p* = 0.0075) and increased diarrhea in both TKI monotherapy (3.50%) and dual ICPI arms (2.49%, *p* = 0.0027). Thus, proactive toxicity monitoring tailored to the specific regimen is required, including gastrointestinal and hematological surveillance.

### 4.4. Head-to-Head Comparison of FDA-Approved First-Line ICPI Regimens

A critical subgroup analysis restricted to 1064 patients enrolled in the three landmark trials of FDA-approved first-line ICPI regimens, namely STRIDE (DurvaTreme, *n* = 393), IMbrave150 (AtezoBev, *n* = 336), and CheckMate 9DW (NivoIpi, *n* = 335), was conducted to facilitate direct evidence-based comparisons without the confounding inclusion of non-FDA-approved agents. Median age across all arms ranged from 64 to 65 years, with 81–83% of patients being male, reflecting the well-established male predominance of HCC.

The analysis revealed that NivoIpi was associated with the longest median OS at 24 months, followed by AtezoBev at 19 months and DurvaTreme at 16.7 months (*p* = 0.046). The PFS results were similarly ordered, with NivoIpi at 9.3 months, AtezoBev at 6.8 months, and DurvaTreme at 3.8 months (*p* < 0.001).

In contrast, grade 3/4 AEs were lowest with DurvaTreme (25.5%), compared with AtezoBev (42.6%) and NivoIpi (40.9%) (*p* < 0.0001), rendering the DurvaTreme regimen particularly attractive in patients with significant comorbidities, limited performance status, or concerns regarding high-grade toxicity. Fatigue and diarrhea were marginally more frequent with DurvaTreme, whereas hypertension was predominantly associated with AtezoBev. These distinct efficacy and safety profiles have significant implications for clinical decision making, supporting a personalized approach to regimen selection based on patient-specific risk factors, etiology, hepatic reserve, and treatment goals.

The superior OS observed with NivoIpi warrants careful interpretation. The CheckMate 9DW trial enrolled patients between 2020 and 2022 with a median follow-up exceeding 35 months, and the combination targets both PD-1 and CTLA-4, generating robust and potentially durable immune memory responses [20]. The five-year OS update from the HIMALAYA trial demonstrated meaningful long-term survival with DurvaTreme, with approximately 19.6% of patients alive at 5 years compared to 15.1% with sorafenib, highlighting the potential for tail-of-the-curve survival benefit unique to immunotherapy regimens [21]. These durable benefit data provide an additional dimension that median OS alone may not fully capture and emphasize the importance of long-term follow-up in interpreting immunotherapy trial outcomes.

### 4.5. Comparison with Prior Meta-Analyses and Network Meta-Analyses

The present analysis builds upon and extends a growing body of NMAs comparing first-line systemic therapies for advanced HCC. In an NMA of nine RCTs (6600 patients) with a search cutoff of October 2022, Celsa et al. found that ICPI plus anti-VEGF combinations achieved the highest probability of OS benefit at 30 months, with AtezoBev and sintilimab plus IBI305 holding the top two rankings (88% and 95% probability, respectively, of being most effective) [38]. Importantly, that study also introduced a safety and efficacy net benefit framework, finding that ICPI plus TKI combinations, specifically camrelizumab plus apatinib, carried a substantially higher severe AE burden compared with AtezoBev, a finding that aligns closely with our observation of a 69.1% grade 3/4 AE rate in the ICPI plus TKI class [38].

Ciliberto et al. similarly conducted an NMA and found that camrelizumab plus rivoceranib ranked highest for OS, lenvatinib plus pembrolizumab for PFS, and sintilimab plus bevacizumab for objective response rate [39]. In an NMA of 15 RCTs (11,796 patients), Liu et al. found that sintilimab plus bevacizumab biosimilar (HR 0.57, 95% CI 0.43–0.75) and camrelizumab plus rivoceranib (HR 0.56, 95% CI 0.41–0.66) demonstrated the largest OS improvements versus sorafenib, while the most cost-effective agent in the Chinese healthcare context was tislelizumab [40].

Most recently, Li et al. conducted an NMA of 17 RCTs (10,322 patients) that incorporated data available through August 2024, confirming the consistent superiority of ICPI-based combinations over TKI monotherapy [41]. Li Q et al. conducted an etiology-stratified NMA incorporating 24 RCTs (13,572 patients). The study further showed that in HBV-related HCC, sintilimab plus bevacizumab biosimilar and AtezoBev led OS rankings, whereas ICPI plus TKI combinations ranked highest for PFS, a finding that partially corroborates the class-level PFS advantage we observed for ICPI plus TKI (HR 0.79, 95% CI 0.73–0.87) [42].

Our analysis extends all of these prior NMAs in three important ways. First, we incorporate data from trials published after the search cutoffs of all prior reviews, most critically CheckMate 9DW [20], APOLLO [24], and HEPATORCH [25], none of which were included in any prior NMA. The inclusion of CheckMate 9DW is especially consequential: the emergence of NivoIpi with the strongest OS signal in the FDA-approved head-to-head subset (median OS 24.08 months, HR 0.70 versus lenvatinib) is a finding that cannot be captured by any prior analysis. Second, our use of IPD reconstruction from Kaplan–Meier curves rather than aggregate hazard ratios enable more granular time-to-event comparisons and avoids reliance on the proportional hazards assumption embedded in conventional aggregate NMA. Third, our pooled grade 3/4 AE meta-analyses provide quantitative toxicity rates across specific AE subtypes (hypertension, platelet abnormalities, anemia, hepatotoxicity), allowing a level of safety and efficacy trade-off analysis that most prior NMAs have either omitted or addressed only in summary form.

### 4.6. Safety Profile and Adverse Event Patterns

Across all subgroup analyses, distinct and clinically meaningful safety profiles emerged for each treatment strategy. The ICPI plus TKI regimen was consistently associated with the highest burden of grade 3/4 AEs, driven by hypertension, platelet abnormalities, and diarrhea, all of which can be mechanistically attributable to the concurrent TKI component [43]. The high incidence of grade 3/4 AEs in the ICPI plus TKI arm is clinically notable and may necessitate dose reductions, treatment interruptions, or discontinuation, potentially attenuating long-term efficacy benefits. The fact that OS with ICPI plus TKI was comparable to dual ICPI and ICPI plus bevacizumab despite this substantially higher toxicity burden further supports the notion that regimen selection should prioritize safety and tolerability where efficacy outcomes are equivalent.

Dual ICPI demonstrated the lowest grade 3/4 AE rate among immunotherapy combination arms (32.7%), with the predominant immune-related AEs being hepatotoxicity and bilirubin elevation, which are predictable class effects of CTLA-4 inhibition.

The ICPI plus bevacizumab arm was associated with proteinuria, hypertension, and platelet abnormalities attributable to bevacizumab, along with immune-mediated events related to the checkpoint inhibitor component. These overlapping but distinct toxicity patterns require integrated, multidisciplinary toxicity surveillance adapted to each regimen.

### 4.7. Methodological Strengths and Limitations

Several methodological strengths merit acknowledgment. The use of IPD reconstruction from Kaplan–Meier curves is a validated approach that permits time-to-event comparisons across trials using different comparators, enabling a more granular and robust synthesis than traditional aggregate meta-analyses. The inclusion of multiple subgroup analyses, with each one addressing a distinct and clinically relevant comparison, enhances the comprehensiveness and applicability of the findings. The large overall sample size provides adequate statistical power to detect meaningful differences across treatment arms.

However, several limitations must be acknowledged. First, cross-trial comparisons are inherently susceptible to between-study heterogeneity, including differences in patient populations, etiology of HCC (hepatitis B versus C versus nonviral), geographic enrollment, Child–Pugh class distribution, and prior treatment history. Such heterogeneity may have influenced the observed treatment effects across studies. In addition, variations in the proportion of patients with macrovascular invasion or extrahepatic disease, follow-up duration, outcome assessment schedules, and adverse event reporting may have affected the comparative evaluation of efficacy and safety outcomes. Therefore, the findings should be interpreted in the context of these potential sources of variability.

Second, the use of IPD reconstruction, while validated, introduces a degree of approximation compared to actual individual patient datasets. Third, the risk of publication bias, particularly in favor of positive trials, cannot be fully excluded. Despite these limitations, the convergent findings across multiple subgroup analyses, with each one conducted independently and addressing different clinically relevant questions, strengthen the overall validity and internal consistency of the conclusions.

Future research should also explore the role of artificial intelligence-based approaches in treatment selection, prognostic stratification, and personalized therapeutic decision making for patients with HCC. As AI technologies continue to evolve, they may complement clinical judgment by integrating complex clinical and molecular data to support precision oncology [44].

### 4.8. Clinical Implications

The findings of the present meta-analysis carry several important clinical implications. For patients with advanced HCC who are eligible for immunotherapy, the choice among first-line ICPI-based regimens should be individualized based on a comprehensive assessment of hepatic function, comorbidity profile, ECOG performance status, vascular invasion risk, and patient preferences regarding route and frequency of administration.

NivoIpi emerges as the regimen with the strongest OS signal among FDA-approved options, making it an attractive choice for patients with good performance status, adequate hepatic reserve, and high disease burden where maximizing survival is the primary objective. The STRIDE regimen DurvaTreme offers a compelling alternative for patients in whom toxicity minimization is paramount, including those with limited comorbidity tolerance or who require intravenous administration without the practical challenges of co-administering bevacizumab. AtezoBev remains a well-established option with the most mature long-term safety data and robust evidence base, particularly suitable for patients without contraindications to bevacizumab, such as recent bleeding events or uncontrolled hypertension.

The ICPI plus TKI strategy, while offering comparable OS to other combination regimens, is associated with substantially higher toxicity and may be best reserved for selected patients who have previously demonstrated tolerance to other regimens or in contexts where intravenous immunotherapy administration is logistically challenging and an oral TKI component is preferred.

### 4.9. Treatment Selection Considerations

#### 4.9.1. Hepatic Function (Child–Pugh Score)

Hepatic reserve is the most critical determinant of systemic therapy eligibility in HCC. All major phase 3 trials were conducted predominantly in Child–Pugh A patients, and the efficacy and safety data from the present analysis apply most directly to this population. Child–Pugh B patients were largely excluded or represented a minor subgroup in the included trials. In Child–Pugh B patients, bevacizumab poses an elevated risk of hepatic decompensation and variceal hemorrhage due to its antiangiogenic mechanism impairing hepatic regeneration; dual ICPI or TKI monotherapy may be more appropriate if systemic therapy is considered at all. Immune-mediated hepatotoxicity from ICPI regimens particularly with CTLA-4 blockade carries additional risk in patients with compromised hepatic reserve, and baseline liver function should be closely monitored throughout treatment. For Child–Pugh C patients, systemic therapy is generally not recommended.

#### 4.9.2. Bleeding Risk (Bevacizumab-Containing Regimens)

Bevacizumab inhibits VEGF-A, thereby impairing angiogenesis and vascular maintenance. This creates specific bleeding risks in patients with HCC, a disease frequently complicated by portal hypertension, esophageal varices, and coagulopathy. All bevacizumab-containing regimens in the present analysis (AtezoBev, ToriBev, SintiBev) require upper endoscopy to screen for and treat high-risk varices prior to treatment initiation, consistent with the IMbrave150 trial protocol. Patients with recent (within 6 months) GI bleeding, grade ≥2 hemoptysis, therapeutic anticoagulation, or uncontrolled hypertension should be considered for alternative ICPI regimens (dual ICPI) or TKI monotherapy. In our analysis, the ICPI plus bevacizumab class was associated with platelet abnormalities in 5.45% of patients at grade 3/4, reinforcing the need for hematological monitoring.

## 5. Conclusions

This network meta-analysis of reconstructed individual patient data from phase 3 randomized controlled trials demonstrates that ICPI-based combination therapies provide superior overall survival compared with tyrosine kinase inhibitor monotherapy as first-line treatment for advanced unresectable HCC. From a treatment class perspective, dual ICPI combinations offered the most favorable efficacy–safety balance. ICPI-based combinations consistently outperformed TKI monotherapy in survival outcomes. Among available regimens, individualized treatment selection is recommended based on hepatic function, bleeding risk, autoimmune comorbidities, performance status, and patient preferences. Future research should focus on biomarker-driven strategies, including validation of PD-L1 expression, tumor mutational burden, and immune microenvironment signatures, as well as the influence of HCC etiology on treatment benefit. Prospective head-to-head randomized trials comparing different ICPI combinations are warranted to establish definitive comparative efficacy and safety.

## Figures and Tables

**Table 1:** Summary of clinical trials included in the study.

Study Name	Year	Country	Intervention	Comparator	Reference
CheckMate 9DW	2025	Asia, Europe, North America, South America, Australia	Nivolumab + Ipilimumab	Lenvatinib/Sorafenib	[20]
IMbrave150	2025	International (17 countries)	Atezolizumab + Bevacizumab	Sorafenib	[16]
HIMALAYA	2025	USA, Italy	Tremelimumab + Durvalumab	Sorafenib	[21]
CARES-310	2023	International (13 countries)	Camrelizumab + Rivoceranib	Sorafenib	[23]
APOLLO	2025	China	Anlotinib + Penpulimab	Sorafenib	[24]
HEPATORCH	2025	China, Taiwan, Singapore	Toripalimab + Bevacizumab	Sorafenib	[25]
ORIENT-32	2021	China	Sintilimab + Bevacizumab biosimilar	Sorafenib	[26]
LEAP-02	2023	International	Lenvatinib + Pembrolizumab	Lenvatinib	[34]
REFLECT	2018	International	Lenvatinib	Sorafenib	[35]

**Table 2:** Cox proportional hazards model for OS and PFS between different ICPI combinations and control (TKI).

	OS (HR)				PFS (HR)			
Characteristic	*N*	HR	95% CI	*p*-Value	*N*	HR	95% CI	*p*-Value
**Class**	6161				6161			
TKI		—	—			—	—	
ICPI mono		0.95	0.78, 1.14	0.57		0.97	0.84, 1.11	0.63
ICPI + Bev		0.70	0.61, 0.81	<0.001		0.99	0.87, 1.13	0.89
ICPI + TKI		0.76	0.68, 0.85	<0.001		0.69	0.62, 0.78	<0.001
ICPI duplet		0.76	0.67, 0.85	<0.001		0.86	0.76, 0.96	<0.001

Abbreviations: CI = confidence interval, HR = hazard ratio.

**Table 3:** Grade 3/4 adverse events across different treatment classes.

Adverse Events	ICPI + Bev	TKI	ICPI Duplet	ICPI	ICPI + TKI	*p*-Value
Fatigue	0.6	1.7	0.7	0.3	1.4	0.407
Hypertension	8.8	8	0.4	1	26.1	<0.001
Bilirubin	2.9	2.6	0.6	1.8	4.3	0.003
Hypothyroidism	0	0	0	0	0.1	1
Diarrhea	0.8	3.9	2.5	1.5	3.8	<0.001
ALT Increase	1.5	2.1	3.6	3.1	5.4	0.053
AST Increase	2.7	4.1	5.6	6.7	7.9	0.073
Hand-foot skin reaction	0	7.4	0	0	4.9	0.946
Other rash	0.2	0.7	1.7	0.3	0.9	0.030
Decreased appetite	1.2	2	0.8	0.5	1.8	0.192
GGT increased	2	2.5	2.1	1.8	4.1	0.836
Nausea	0.3	0.6	0	0	0.4	0.985
Anemia	5.6	2.2	2.8	2.3	1.8	0.116
Hypokalemia	1.1	1.6	1	0.8	2.5	0.

Abbreviations: ICPI + Bev, immune checkpoint inhibitor plus bevacizumab; TKI, tyrosine kinase inhibitor; ICPI duplet, dual immune checkpoint inhibitor therapy; ICPI, immune checkpoint inhibitor monotherapy; ICPI + TKI, immune checkpoint inhibitor plus tyrosine kinase inhibitor; ALT, alanine aminotransferase; AST, aspartate aminotransferase; GGT, gamma-glutamyl transferase.

**Table 4:** Median OS and median PFS for all the approved first-line treatments.

	OS (Months)	PFS (Months)		
Characteristic	Median OS (95% CI)	*p*-Value ^1^	Median PFS (95% CI)	*p*-Value ^1^
Overall	18.37 (17.17, 19.15)		6.762 (6.247, 7.346)	
Treatment		<0.001		<0.001
Lenvatinib	16.98 (15.63, 18.93)		7.356 (7.243, 8.384)	
AtezoBev	19.09 (17.06, 23.60)		6.866 (5.768, 8.575)	
NivoIpi	24.08 (19.50, 30.29)		9.333 (6.448, 10.61)	
DurvaTreme	16.75 (14.18, 20.26)		3.840 (3.673, 5.426)	

^1^ Log-rank test.

**Table 5:** Cox proportional hazards model for OS and PFS between the approved first-line treatment and lenvatinib.

	OS (HR)				PFS (HR)			
Characteristic	*N*	HR	95% CI	*p*-Value	*N*	HR	95% CI	*p*-Value
	2210				1941			
Lenvatinib		—	—			—	—	
AtezoBev		0.86	0.73, 1.02	0.075		0.93	0.81, 1.07	0.3
NivoIpi		0.70	0.60, 0.82	<0.001		0.73	0.63, 0.85	<0.001
DurvaTreme		0.88	0.77, 1.01	0.077		1.26	1.10, 1.43	<0.001

Abbreviations: CI = Confidence Interval, HR = Hazard Ratio.

## Data Availability

The datasets analyzed during the current study are available from the corresponding author upon reasonable request.

## Supplementary Materials

The following supporting information can be downloaded at: https://www.mdpi.com/article/10.3390/cancers18132118/s1, Supplemental S1: List of R packages used in the analysis and the manuscript. Figure S1. Risk of bias analysis of included studies. Table S1. Number of patients across different treatment groups, classes, and trials. Table S2. Total grade 3/4 and the most common G3/4 adverse events between different treatment classes (extended). Table S3. The most common adverse events between first-line treatments. Supplemental S2: Preferred Reporting Items for Systematic Reviews and Meta-Analyses Extension for Scoping Reviews (PRISMA-ScR) Checklist. Reference [45] is cited in the Supplementary Materials.
