# Supplementary material for: Comprehensive Safety and Efficacy Evaluation of Immunotherapy Combination Approaches Versus Tyrosine Kinase Inhibitor Monotherapy as First-Line Treatment of Hepatocellular Carcinoma: A Network and Individual Patient Data (IPD) Meta-Analysis
Source: Cancers (Basel). 2026 Jun 30;18(13):2118. doi: 10.3390/cancers18132118 (PMC13359836; doi:10.3390/cancers18132118)
Supplement: Supplementary file 1 [file cancers-18-02118-s001.zip › Supplementary material-for revision.pdf]

cancers

MDPI

Supplementary Materials: Comprehensive Safety and Efficacy Evaluation of Immunotherapy Combination Approaches Versus Tyrosine Kinase Inhibitor Monotherapy as First-Line Treatment of Hepatocellular Carcinoma: A Network and Individual Patient Data (IPD) Meta-Analysis

Abdullah Esmail, Yazan Hamdaneh Nour Mustafa, Ebtesam Al-Najjar, Zaid Alabed, Hikmat Abdel-Razeq, Asem Mansour and Maen Abdelrahim

Supplemental S1

List of R packages used in the analysis and the manuscript:

readxl

pacman

readr

ggplot2

ggsurvfit

survminer

ggthemes

stringr

rio

tidyr

vtable

survival

tidyverse

ggfortify

gt

gtsummary

meta

netmeta

metafor

glmtoolbox

scales

emmeans

coxme

PRISMA2020

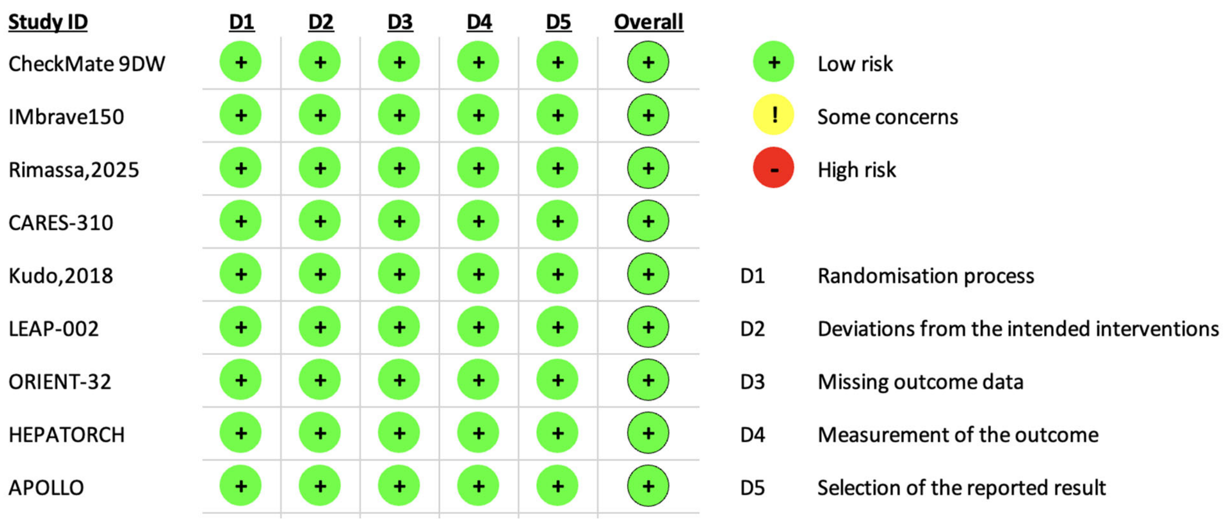

Figure S1. Risk of bias analysis of included studies.

Table S1. Number of patients across different treatment groups, classes, and trials.

Characteristic	N = 6,161[1]
**Treatment**	
Sorafenib	1,920 (31%)
AnloPenp	433 (7.0%)
AtezoBev	336 (5.5%)
CamreRivo	272 (4.4%)
Durvalumab	389 (6.3%)
Lenvatinib	1,146 (19%)
LenvaPembro	395 (6.4%)
NivoIpi	335 (5.4%)
SintiBev	380 (6.2%)
DurvaTreme	393 (6.4%)
ToriBev	162 (2.6%)
**Study**	
APOLLO	649 (11%)
CARES310	543 (8.8%)
CHECKMATE9DW	652 (11%)
HEPATORCH	326 (5.3%)
HIMALAYA	1,171 (19%)
IMBRAVE150	501 (8.1%)
Kudo et al, 2018	954 (15%)
LEAP-002	794 (13%)
ORIENT-32	571 (9.3%)
**Class**	
TKI	3,066 (50%)
ICPI	389 (6.3%)
ICPI + Bev	878 (14%)
ICPI + TKI	1,100 (18%)
ICPI Duplet	728 (12%)

Table S2. Total grade 3/4 and the most common G3/4 adverse events between different treatment classes (extended).

Adverse Events	ICPI + Bev	TKI	ICPI Duplet	ICPI	ICPI + TKI	*p* Value
**Total Grade 3/4**	38.6	50.1	32.7	12.9	69.1	< 0.001
**Fatigue**	0.6	1.7	0.7	0.3	1.4	0.407
**Hypertension**	8.8	10.2	0.4	1	23	< 0.001
**Bilirubin**	2.9	2.6	0.6	1.8	4.3	0.00391
**Diarrhea**	0.8	3.9	2.5	1.5	3.8	< 0.001
**ALT increase**	1.5	2.1	3.6	3.1	5.4	0.0526
**AST increase**	2.7	4.1	5.6	6.7	7.9	0.0731
**Hand-foot skin reaction**	0	7.4	0	0	4.9	< 0.001
**Rash**	0.2	0.7	1.7	0.3	0.9	0.0302
**Platelets decreased**	5.4	2.1	0.4	NA	7.8	0.00194
**Proteinuria**	2.9	1.7	0	NA	3.2	0.719
**Appetite decreased**	1.2	2	0.8	0.5	1.8	0.192
**GGT increased**	2	2.5	2.1	1.8	4.1	0.836
**Nausea**	0.3	0.6	0	0	0.4	0.985
**Anemia**	5.6	2.2	2.8	2.3	1.8	0.116
**Hypokalemia**	1.1	1.6	1	0.8	2.5	0.292
**Liver dysfunction**	0.9	0.9	0	NA	NA	1
**Fever**	0	0.1	0.3	1.8	NA	0.0423

Table S3. The most common adverse events between first-line treatments.

Adverse Event	Atezo-Bev	NivoIpi	DurvaTreme	Lenvatinib	*p* Value
**Total Grade 3/4**	42.6	40.9	25.4	66.3	< 0.001
**Fatigue**	0.4	0.2	1.9	2.6	0.0405
**Hypertension**	12	0	1.8	20.3	< 0.001
**Bilirubin**	NA	0.3	0.8	4.4	0.00212
**Hypothyroidism**	0	0	0	0	1
**Diarrhea**	0.9	1.2	4.4	4	0.00814

https://doi.org/10.3390/xxxxx

**ALT**	2.5	4.8	2.6	3	0.282
**AST**	5.2	6	5.2	4.7	0.833
**Hand-foot skin reaction**	0	0	0	3.1	< 0.001
**Rash other than**	0	1.8	1.5	0	0.995
**Platelets**	2.8	0.4	NA	4.9	0.073
**Proteinuria**	4	0	NA	5.6	0.526
**Appetite**	0.9	0.3	1.3	3.9	0.00127

https://doi.org/10.3390/xxxxx
